# The failure of protein cancer biomarkers to reach the clinic: why, and what can be done to address the problem?

**DOI:** 10.1186/1741-7015-10-87

**Published:** 2012-08-09

**Authors:** Eleftherios P Diamandis

**Affiliations:** 1 Department of Pathology and Laboratory Medicine, Mount Sinai Hospital, Toronto, ON, Canada; 2 Department of Clinical Biochemistry, University Health Network, Toronto, ON, Canada; 3 Department of Laboratory Medicine and Pathobiology, University of Toronto, Toronto, ON, Canada

**Keywords:** biomarker failures, biomarker validation, cancer biomarkers, false discovery, improved biomarkers, proteomics

## Abstract

There is a plethora of published cancer biomarkers but the reality is that very few, if any, new circulating cancer biomarkers have entered the clinic in the last 30 years. I here try to explain this apparent oxymoron by classifying circulating cancer biomarkers into three categories: fraudulent reports (rare); true discoveries of biomarkers, that then fail to meet the demands of the clinic; and false discoveries, which represent artifactual biomarkers. I further provide examples of combinations of some known cancer biomarkers that can perform well in niche clinical applications, despite individually being not useful.

## Background

There is wide debate recently as to why very few, if any, new circulating cancer biomarkers have entered the clinic in the last 30 years. The vast majority of clinically useful cancer biomarkers were discovered between the mid-1960s (for example, carcinoembryonic antigen, CEA) and the early 1980s (for example, prostate-specific antigen (PSA) and carbohydrate antigen 125 (CA125)). It is true that major investments by both academia and industry have been made in this area of investigation but with very little return. One wonders why this happens in an era of spectacular technological advances. Some argue that it is because the problem is very complex and underestimated; others blame reasons such as a lack of understanding of the pathobiology of cancer, not enough funding, use of inappropriate samples for discovery and validation, methodological limitations, and so on [1,2].

Here, I will provide a brief and simplified analysis as to why this may be happening. We should keep in mind that the high failure rates in the biomarker field are no different from those of therapeutics. But there is an important difference. Therapeutics leading to relatively small improvements in patient survival (weeks to months) are likely to be marketed, while diagnostics with relatively small improvements in patient diagnosis or prognosis will likely fall by the wayside. Hence, similar advances in therapeutics and diagnostics can be hailed as 'successes' in the former and 'failures' in the latter.

## Why do most biomarkers fail to reach the clinic?

**Figure 1:**
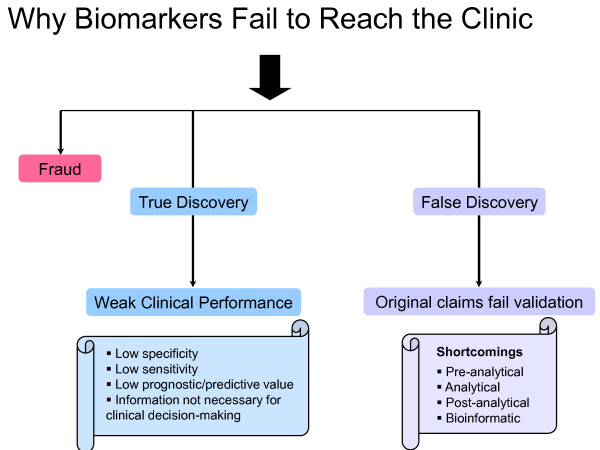
**Summary of reasons for biomarker failure to reach the clinic**. Fraud is a very rare reason for biomarker failures; most biomarkers represent true discoveries but their clinical characteristics are not good enough to be used at the clinic.

One way of analyzing the apparent failures in diagnostics is by classifying them into three distinct categories (Figure 1). The first category of failing biomarkers includes those that are based on fraudulent publications. These are quite rare and, despite some highly-publicized cases [3], fraud is not the major reason for failing biomarkers.

The second and largest category of biomarkers that never reach the clinic (and counted as failures) includes those that have been discovered and validated by using robust and reliable techniques (true discovery). These biomarkers have successfully gone through the process of discovery and validation, with reproducible and concordant results between studies, from early to late stages. However, these biomarkers fall short in their ability to contribute decisively to patient care, except for providing some incremental, but clinically not essential, information. For example, the urokinase plasminogen activator/plasminogen activator inhibitor 1 (uPA/PAI 1) combination of biomarkers has long been regarded as a prognostic indicator of breast carcinoma, and many retrospective and prospective studies and meta-analyses have confirmed their prognostic value [4]. However, they have not been widely adopted, especially in North America, despite availability of excellent ELISA methodologies for their measurement, because clinicians do not seem to find this information necessary in deciding how to treat their patients. Rather, they decide on treatment options without this information, thus saving costs. Clinicians usually prefer to over-treat some patients, instead of using prognostic biomarkers with less than perfect prediction. Imperfect prognostic biomarkers could spare a fraction of patients from over-treatment (true positives), but at the cost of not treating some patients who could benefit from treatment (false negatives). Another example is the tumor suppressor p53. A search in PubMed for the term 'p53 AND breast cancer prognosis' identifies 1470 papers, with the vast majority confirming the prognostic value of p53, despite its sparse use at the clinic, if any, due to the reasons mentioned above (imperfect or weak prognostic value).

Other examples in this category include those biomarkers that have been discovered and validated thoroughly by industry, and although found to have some use in clinical prediction, the strength of their predictive ability is not enough to persuade clinicians to use them, or clinical practice guideline developers to recommend them. An example is the novel ovarian cancer biomarker, B7-H4 (discovered by diaDexus Inc., San Francisco, CA, USA), which was validated for its ability to diagnose ovarian cancer [5]. Recently, an independent group confirmed the diagnostic ability of this biomarker, but also demonstrated that it is not better than the classical biomarker, CA125 [6]. Consequently, the company that discovered it, in the absence of a clear clinical utility, decided not to market it. It is quite expensive to conduct the necessary clinical trials to obtain approval from the Food and Drug Administration (FDA). There are numerous examples of this sort in the literature, that is, of reasonable and working biomarkers that fall short of fulfilling a clear clinical need and thus unlikely to be profitable if marketed.

There are also numerous examples of biomarkers which have good sensitivity (>70% at 90% to 95% specificity) in detecting late carcinoma but poor sensitivity in detecting disease in asymptomatic patients, especially at the extremely high specificity required for screening (for example, >99.5%, as is the case with ovarian carcinoma) [6]. Consequently, none of the available ovarian cancer biomarkers are suitable for screening, and it may be unlikely that we will find any that can perform at these clinically dictated and highly demanding specifications (for example, >80% sensitivity for early and asymptomatic disease, at ≥ 99.5% specificity; to achieve a reasonable positive predictive value of ≥10%).

From this discussion, it can be concluded that a very large number of candidate biomarkers have been discovered, and have been confirmed by reliable methods to provide diagnostic, prognostic or predictive information in certain groups of patients. Unfortunately, this information could not be translated into action for better patient management and outcomes. So, work on biomarkers is continuing. However, these biomarkers are not recommended in practice guidelines for use in the diagnosis or treatment of cancer [7], despite their statistically significant (but clinically not useful) diagnostic, predictive or prognostic information. Some examples of well-validated biomarkers and their possible reason of failure at the clinic are outlined in Table 1.

**Table 1:** Why well-validated biomarkers still fail to reach the clinic?

Clinical application	Reason to Fail	Example
Population screening and diagnosis	• screening does not save lives; overdiagnosis/overtreatment	PSA and prostate cancer screening
	• too many false positives leading to unnecessary and invasive confirmatory procedures	• CA 125 for ovarian cancer screening
	• marker not profitable if marketed	• B7-H4 for ovarian cancer diagnosis
Prognosis	• weak prognostic value; clinicians prefer to overtreat instead of undertreat	• p53 and uPA/PAI1 for breast cancer
	• no effective therapy available	• CA 19.9 for pancreatic cancer
Monitoring	• no therapy for relapsing disease	• CA125 for detection of early relapse of ovarian cancer
Evaluating therapeutic response	• many false positives and false negatives	• CA 15.3 for breast cancer

CA: carbohydrate antigen; PSA: prostate-specific antigen

There is another group of biomarkers which may initially look highly promising (or even revolutionary) but for which shortcomings have been identified, either at the discovery or validation phase. For example, in my previous commentary [8], I identified pre-analytical, analytical and post-analytical shortcomings of many published biomarkers, which could invalidate the original performance claims. This group of biomarkers should be considered as 'false discovery'. They will not reach the clinic because the original performance claims could not be independently reproduced in subsequent validation studies.

## Future prospects: how do we overcome failures?

In light of the analysis above, it can be concluded that the failure of the myriad of biomarkers to reach the clinic, excluding fraud, is either due to inadequate performance in a clinical setting or to false discovery. There are at least two ways to improve this situation. For those biomarkers with a well-validated performance, but which are not good enough for clinical use, it may be possible to identify clinical scenarios for which the markers could still help, in combination with other clinical or biomarker data. For example, human epididymis protein 4 (HE4), a new ovarian cancer biomarker, is not superior to CA125 for diagnosis of ovarian carcinoma [6] but is more specific. A combination of CA125 and HE4, through an algorithm, was found to be useful in the investigation of malignant versus benign pelvic masses [9]. Another test combines serum CA125 with a few other proteomic biomarkers which, individually, are not useful [10]. Both tests have recently received FDA approval (2011; 2009, respectively) for this specific application, thus reaching the clinic, in combinations, but not individually. There are examples of similar applications of otherwise not very informative diagnostic biomarkers, such as in the investigation of a computed tomography-positron emission tomography-identified indeterminate lung masses, for which a combination of already-known (but individually not useful) biomarkers may help [11]. Similar examples of 'niche unmet needs' include the identification of biomarkers to assess the risk of malignancy for thyroid nodules, specifically those with indeterminate results after standard thyroid biopsy; identification of biomarkers to improve on PSA's specificity in screening (such as prostate cancer gene 3) [12]; or, following diagnosis of prostate cancer, identification of biomarkers that discern between indolent versus aggressive prostate cancers. Table 2 summarizes these niche unmet needs.

**Table 2:** Niche applications of combinations of biomarkers with Food and Drug Administration approval or with potential in the future (unmet needs)

Clinical application	Biomarkers	Status
Investigation of pelvic mass	• CA 125 and HE4 (+ ROMA)^a^ • CA 125 and 7 proteomic markers (+ Danish-Index)^b^	• FDA-approved (2011) • FDA-approved (2009)
Improve PSA specificity in screening	• Serum PSA and urine PCA-3 and TMPRSS2-ERG fusions^c^	• Pending FDA approval
Separate indolent from aggressive prostate cancer	• PTEN loss^c^ • TMPRSS2-ERG fusions^3^	• More research necessary^e^
Assess risk of malignancy of thyroid nodules with indeterminate results on biopsy	• HBME-1, Galectin-3 and CK19^d^	• More research necessary
Assess risk of malignancy of CT (± PET) imaging-identified indeterminate lung masses	• CEA, CYFRA 21-1, SCC, CA15.3, Pro-GRP, NSE	• More research necessary

^a ^See [9] for details; ^b ^see [10] for details; ^c ^see [12,15] for details; ^d ^see [16,17] for details. HBME-1 is an antimesothelial cell monoclonal antibody; ^e ^PCA-3 was approved by FDA (2012) to help determine the need for repeat prostatic biopsy in men who had a previous negative biopsy. See also [18]. CA: carbohydrate antigen; CT: computed tomography; CK19: cytokeratin 19; CYFRA 21-1: cytokeratin fragment 21-1; FDA: Food and Drug Administration; HBME-1: human bone marrow endothelial 1; HE4: human epididymis protein 4; NSE: neuron-specific enolase; PET: positron emission tomography; pro-GRP: pro-gastrin releasing peptide; PCA3: prostate cancer gene 3; PSA: prostate-specific antigen; PTEN: phosphatase and tensin homolog; ROMA: risk of ovarian malignancy algorithm; TMPRSS2-ERG: transmembrane protease, serine 2 *(*TMPRSS2*) *and v-ets erythroblastosis virus E26 oncogene homolog (avian) *(*ERG*)*

Another critical question is what could be done to avoid false discovery. Recommendations for this problem have been proposed elsewhere [2,8] and include understanding and avoidance of pre-analytical shortcomings; careful study design to avoid bias [13,14]; use of analytical methodologies that are sensitive, specific and precise; selecting appropriate samples (in numbers and quality) and patient subgroups for validation; and application of robust and rigorous statistics to avoid data over-fitting.

## Conclusion

In theory, biomarkers can serve many clinical needs from risk stratification to prognosis, screening, diagnosis, monitoring, patient subclassification, assessment of drug toxicity and prediction of therapeutic response. To bring biomarkers to the clinic, it is mandatory to show a useful clinical application that is supported by the validation data. Only then will diagnostic companies invest the necessary (and very significant) funds to conduct multicenter clinical trials to show efficacy and receive FDA approval. In conclusion, between the two groups of biomarkers (false discovery and true discovery), the former can be considered a failure but the latter should not. Distinguishing between these two categories is important, since true discovery is based on good science and statistically significant and reproducible data, while false discovery is based on bad science. As shown with examples, some fruits of true discovery can be combined to design incremental but clinically useful and FDA-approved improvements (Table 2). Eventually, the time will likely come when biology and technology will advance sufficiently to catalyze the needed and much anticipated leap in cancer biomarkers.

## Competing interests

The author declares that they have no competing interests.

## Author's information

Dr. Diamandis currently serves as Division Head of Clinical Biochemistry at Mount Sinai Hospital and Biochemist-in-Chief at University Health Network, and is Professor and Head of Clinical Biochemistry, University of Toronto, Ontario, Canada. His research activities revolve around the discovery and validation of cancer biomarkers, proteomics, mass spectrometry and translational medicine.

## Pre-publication history

The pre-publication history for this paper can be accessed here:

http://www.biomedcentral.com/1741-7015/10/87/prepub

## References

[B1] Buchen L Missing the mark. Why is it so hard to find a test to predict cancer? Nature 2011 471 428 432 10.1038/471428a 21430749

[B2] Hanash SM Why have protein biomarkers not reached the clinic? Genome Med 2011 3 66 10.1186/gm282 22030259 PMC3239228

[B3] Reich ES Cancer trial errors revealed Nature 2011 469 139 140 10.1038/469139a 21228842

[B4] Kantelhardt EJ Vetter M Schmidt M Veyret C Augustin D Hanf V Meisner C Paepke D Schmitt M Sweep F von Minckwitz G Martin PM Jaenicke F Thomssen C Harbeck N Prospective evaluation of prognostic factors uPA/PAI-1 in node-negative breast cancer: phase III NNBC3-Europe trial (AGO, GBG, EORTC-PBG) comparing 6 × FEC versus 3 × FEC/3 × Docetaxel BMC Cancer 2011 11 140 10.1186/1471-2407-11-140 21496284 PMC3089797

[B5] Simon I Zhuo S Corral L Diamandis EP Sarno MJ Wolfert RL Kim NW B7-h4 is a novel membrane-bound protein and a candidate serum and tissue biomarker for ovarian cancer Cancer Res 2006 66 1570 1575 10.1158/0008-5472.CAN-04-3550 16452214

[B6] Cramer DW Bast RC Berg CD Diamandis EP Godwin AK Hartge P Lokshin AE Lu KH McIntosh MW Mor G Patriotis C Pinsky PF Thornquist MD Scholler N Skates SJ Sluss PM Srivastava S Ward DC Zhang Z Zhu CS Urban N Ovarian cancer biomarker performance in prostate, lung, colorectal and ovarian cancer screening trial specimens Cancer Prev Res 2011 4 365 374 10.1158/1940-6207.CAPR-10-0195 PMC3085251 21372036

[B7] Diamandis EP Hoffman BR Sturgeon CM National Academy of Clinical Biochemistry laboratory medicine practice guidelines for the use of tumor markers Clin Chem 2008 54 1935 1939 10.1373/clinchem.2008.105494 18801938

[B8] Diamandis EP Cancer biomarkers: can we turn recent failures into success? J Natl Cancer Inst 2010 102 1462 1467 10.1093/jnci/djq306 20705936 PMC2950166

[B9] Molina R Escudero JN Augé JM Filella X Foj L Torné A Lejarcegui J Pahisa J HE4 a novel tumour marker for ovarian cancer: comparison with Ca125 and ROMA algorithm in patients with gynaecological diseases Tumour Biol 2011 32 1087 1095 10.1007/s13277-011-0204-3 21863264 PMC3195682

[B10] Hogdall C Fung ET Christensen IJ Nedergaard L Engelholm SA Petri AL Risum S Lundvall L Yip C Pedersen AT Hartwell D Lomas L Høgdall EV A novel proteomic biomarker panel as a diagnostic tool for patients with ovarian cancer Gynecol Oncol 2011 123 308 313 10.1016/j.ygyno.2011.07.018 21855971

[B11] Molina R Holdenrieder S Auge JM Schalhorn A Hatz R Stieber P Diagnostic relevance of circulating biomarkers in patients with lung cancer Cancer Biomark 2010 6 163 178 20660962 10.3233/CBM-2009-0127 PMC12922860

[B12] de la Taille A Irani J Graefen A Chun F de Reijke T Kil P Gontero P Mottaz A Haese A Clinical evaluation of the PCA3 assay in guiding initial biopsy decisions J Urol 2011 185 2119 2125 10.1016/j.juro.2011.01.075 21496856

[B13] Pepe MS Feng Z Janes H Bossuyt PM Potter JD Pivotal evaluation of the accuracy of a biomarker used for classification or prediction: standards for study design J Natl Cancer Inst 2008 100 1432 1438 10.1093/jnci/djn326 18840817 PMC2567415

[B14] Ransohoff DF Bias as a threat to the validity of cancer molecular-marker research Nat Rev Cancer 2005 5 142 149 10.1038/nrc1550 15685197

[B15] Sardana G Dowell B Diamandis EP Emerging biomarkers for the diagnosis and prognosis of prostate cancer Clin Chem 2008 54 1951 1960 10.1373/clinchem.2008.110668 18927246

[B16] Fadda G Rossi ED Raffaelli M Pontecorvi A Sioletic S Morassi F Lombardi CP Zannoni GF Rindi G Follicular thyroid neoplasms can be classified as low- and high-risk according to HBME-1 and Galectin-3 expression on liquid-based fine-needle cytology Eur J Endocrinol 2011 165 447 453 10.1530/EJE-11-0181 21724837

[B17] Nga ME Lin GS Soh CH Kumarasinghe MP HBME01 and CK19 are highly discriminatory in the cytological diagnosis of papillary thyroid carcinoma Diagn Cytopathol 2008 36 550 556 10.1002/dc.20841 18618720

[B18] Presner JR Rubin MA Wei JT Chinnaiyan AM Beyond PSA: the next generation of prostate cancer biomarkers Sci Transl Med 2012 4 127rv3 10.1126/scitranslmed.3003180 PMC3799996 22461644

